# An Audit on the Pre-operative Fasting Time of Trauma-List Orthopaedic Patients at a District General Hospital in Chichester, United Kingdom

**DOI:** 10.7759/cureus.48327

**Published:** 2023-11-05

**Authors:** Hector Perera, Adedoyin Wusu, Alhashash Mohammad, Mahdi Z Qulaghassi, Ali Abdulkarim

**Affiliations:** 1 General Surgery, Royal Sussex County Hospital, Brighton, GBR; 2 Trauma and Orthopaedics, St Richard's Hospital, Chichester, GBR; 3 Trauma and Orthopaedics, Medway Maritime Hospital, Gillingham, GBR

**Keywords:** compliance to guidelines, general anaesthesia, orthopaedics surgery, clincal audit, preoperative fasting

## Abstract

Introduction: Pre-operative fasting of patients awaiting non-emergency surgeries has been a common practice to minimise the risk of vomiting and aspiration at the time of induction of anaesthesia. Current standard guidelines recommend that this fasting time be limited to two hours for clear fluids and six hours for solids and semi-solids, as prolonged fasting has been shown to be harmful to the patient.

Methods: A descriptive cross-sectional study of the fasting times of all adult trauma orthopaedic patients who were operated on under anaesthesia between June 1 and 30, 2023. Fifty patients who met the inclusion criteria were included in the study.

Results: The minimum and maximum fasting times observed for solids and semi-solids were 9 and 24 hours, respectively. The mean fasting time for solids and semi-solids was 15.8 hours. The minimum and maximum fasting times observed for clear fluids were 2 and 20 hours, respectively. The mean fasting time for clear fluids was 10.5 hours. Elderly patients accounted for a significant portion of the patients, with 64% (n=32) being above the age of 70 years.

Conclusion: A significant disparity was noted between the current fasting practices and the recommended standards set out by the Royal College of Nursing, the Association of Anaesthetists of Great Britain and Ireland, the European Society of Anaesthesiology, and the American Society of Anaesthesiologists. The knowledge of pre-operative fasting among the orthopaedic team doctors and the ward nursing staff was found to be inadequate.

## Introduction

It has been common practice to advise patients awaiting non-emergent surgeries to fast prior to surgery. The aim of such practice has mainly been to prevent vomiting, regurgitation, and aspiration of acidic gastric contents at the time of induction of anaesthesia, which can result in significant complications and poor outcomes for patients [1]. In view of this, patients awaiting surgery under general and/or regional anaesthesia have been advised to remain fasting ‘overnight’ or ‘from midnight’ traditionally. This has led to patients being fasted for prolonged periods prior to elective and other non-emergent surgeries. Prolonged fasting is associated with excessive hunger and thirst, resulting in patient distress. It can also lead to dehydration, electrolyte imbalances, hypoglycaemia, post-operative nausea and vomiting, and an increased risk of acute kidney injury (AKI), especially in the elderly population [2,3]. In addition, it has been shown that prolonged starvation can lead to insulin resistance, which in turn may increase the risk of post-operative infections [4,5].

Studies on gastric emptying have shown that clear liquids tend to be cleared from the stomach within one to two hours of ingestion, and solids can take up to- four hours, depending on the nature of the food ingested [6,7]. Hence, it is of little use to starve patients for prolonged periods pre-operatively. In fact, the undesirable effects of prolonged fasting should be a reminder of the importance of adhering to the latest guidelines on pre-operative fasting to make the experience of the surgery a pleasant one for the patient. Most international guidelines now recommend that in adults, a fasting period of two hours for clear liquids and six hours for solids or semi-solids prior to the induction of general or regional (spinal, epidural, or peripheral nerve block) anaesthesia is optimum for elective and non-emergent surgeries [8-10]. This helps to strike a balance between maintaining patient safety with adequate pre-operative fasting while avoiding undue patient discomfort and complications that may arise from over-fasting.

This audit was conducted at a district general hospital in Chichester, United Kingdom, which provides health services to over 200,000 people residing in the Southwest Sussex and East Hampshire areas of the United Kingdom. The main aim of this audit was to assess the pre-operative fasting practices carried out for patients undergoing surgery on the orthopaedic trauma list under general and/or regional anaesthesia. Both upper and lower limb surgeries were included in the study. The objectives of this study were as follows: assess the pre-operative fasting times of these patients for clear fluids and solids/semi-solids; assess the degree of adherence or deviation from the recommended guidelines; evaluate the instructions that had been documented in the notes on pre-operative fasting; and determine the level of knowledge on pre-operative fasting among the doctors in the orthopaedic department and the nursing staff of the orthopaedic ward. As this audit was carried out as a retrospective evaluation of patient records, the determination of the adverse effects of prolonged fasting was not within the scope of this study.

## Materials and methods

The audit was conducted as a retrospective cross-sectional study, covering a period of one month between June 1 and 30, 2023. We used a consecutive sampling technique, where all cases that met the inclusion criteria and were operated on under general and/or regional anaesthesia within the defined study period were included in the audit. The list of patients who had undergone orthopaedic surgery under the trauma list in the above study period was obtained from 'etrauma', which is the online database that is used by the orthopaedic department of the hospital. It has been common practice in the hospital that all surgical patients who come to the theatre are routinely assessed by a nurse in the theatre pre-operative area and are questioned about the time of last clear liquid intake and the time of last meal. These details are recorded in the hospital theatre care pathway document. The time of the induction of anaesthesia and the time of the surgery are also recorded in this document. All patient documents are generally uploaded to the secure hospital online platform 'affinity' following patient discharge. Hence, the theatre care pathway documents were easily accessed and analysed through affinity.

Surgeries that were classified as “immediate” under the National Confidential Enquiry into Patient Death and Outcome (NCEPOD), which required intervention simultaneous with resuscitation for immediate life or limb-threatening conditions, were excluded from the study [11]. Therefore, the study only included the theatre care pathway documents of adults who had required 'urgent' or 'expedited' interventions under the trauma list. Elective surgical cases were not included in the study. The theatre care pathway documents of 50 cases that fit the above criteria within the given study period were assessed in the audit. We also analysed the instructions documented regarding pre-operative fasting in the patient notes. These documents were once again easily accessed through affinity.

Our study included only the trauma list patients, who would generally be admitted to the emergency department and operated on the following day. These patients would often be seen by the anaesthetists only on the morning of the surgery after the daily trauma meeting discussion. Therefore, instructions on pre-operative fasting were given overnight by the orthopaedic team doctors and enforced by the ward nursing staff. Therefore, we conducted an online survey among the orthopaedic team doctors and the nursing staff of the orthopaedic ward to gauge their level of knowledge on pre-operative fasting guidelines. This audit was conducted as a retrospective evaluation of existing medical records for quality assurance purposes. Therefore, we did not require ethics committee approval. The audit was registered with the trust’s clinical audit department.

## Results

From the trauma list surgeries that were carried out during the study period of June 1 to June 30, 2023, a total of 50 patients met the inclusion criteria and were included in the study. Females were the predominant gender, accounting for 58% (n=29) of the study population. Elderly patients accounted for a significant portion of the patients, with 64% (n=32) being above the age of 70 years. Only 16% (n=8) of patients were below 50 years old. Of the study population, 16% (n=8) were on treatment for diabetes mellitus. The demographic characteristics of the study population have been summarised in Table 1.

**Table 1:** Demographic data of the patients operated in trauma list between the June 1 and 30, 2023

		Number of patients (n)	Percentage (%)
Gender	Male	21	42
	Female	29	58
Age	<30	1	2
	30-49	7	14
	50-69	10	20
	70-90	26	52
	>90	6	12
Diabetic status	Yes	8	16
	No	42	84

Neck-femur fracture was the most common indication for surgery among the study population, accounting for 54% (n=27) of the cases. This was expected, given that neck-femur fractures are one of the most common orthopaedic presentations in the elderly. Surgical fixation for ankle fractures was the next commonest, accounting for 12% of patients (n=6), followed by surgical fixation of periprosthetic fractures, which accounted for 6% of patients (n=3) (Table 2).

**Table 2:** Indications for surgery among the patients operated in trauma list between June 1 and 30 ORIF: open reduction and internal fixation

Indication for surgery and the surgical intervention	Number of patients (n)	Percentage (%)
Neck of femur fractures (hemiarthroplasty/total hip replacement/Internal fixation)	27	54
Ankle fractures (ORIF/external fixator)	6	12
Periprosthetic fracture (surgical fixation)	3	6
Wrist fractures (ORIF)	2	4
Tibial plateau fractures internal fixation	2	4
Other	10	20

The minimum and maximum fasting times observed for solids or semi-solids were 9 and 24 hours, respectively. The mean fasting time for solids or semi-solids was 15.8 hours. As much as 74% (n=37) of the patients had been fasting for more than 12 hours, while 94% (n=47) had a fasting time of more than 10 hours for solids and semi-solids. All the patients in the study population had been fasted for solids and semi-solids beyond the recommended six hours (Table 3).

**Table 3:** Fasting time for solid and semi-solid meal in the study population

Fasting time (hours)	Number of patients (n)	Percentage (%)
0–6	0	0
7–9	3	6
10–12	10	20
>12	37	74

The minimum and maximum fasting times observed for clear fluids were 2 and 20 hours, respectively. The mean fasting time for clear fluids was 10.5 hours. It was noted that 72% (n=36) of the patients did not have any clear fluids for more than nine hours prior to the surgery, while 86% (n=43) fasted for more than six hours prior to the surgery for clear fluids. It was noted that 98% (n=49) of the patients had fasted for clear fluids for more than the recommended time of two hours (Table 4).

**Table 4:** Fasting time for clear liquids in the study population

Fasting time (hours)	Number of patients (n)	Percentage (%)
0–2	1	2
3–5	6	12
6–8	7	14
9–12	19	38
>12	17	34

On the evaluation of the documented instructions for pre-operative fasting in the patient notes, we noted that 62% (n=31) of the patients were instructed to be ‘nil by mouth from midnight’, with no distinction made with regard to solids and clear fluids. In 22% (n=11) of the notes, it was documented as ‘nil by mouth’, with no distinction made to the time duration of fasting or with regards to solids and clear fluids. Only 10% (n=5) of the patients were given instructions to fast for solids for six hours and clear fluids for two hours prior to surgery. Pre-operative intravenous fluids were administered to 52% (n=26) of the patients (Table 5).

**Table 5:** Pre-operative fasting instructions documented on the notes and the pre-operative intravenous fluid status

		Number of patients (n)	Percentage (%)
Instructions	Nil by mouth - midnight	31	62
	Nil by mouth (no time specified)	11	22
	Nil by mouth - solids from 2am, clear liquids from 6 am	5	10
	Nil by mouth - 2 am	3	6
Pre-operative IV fluids	Yes	26	52
	No	24	48

We also conducted an online survey among the doctors in the trauma and orthopaedics department (specialty registrars, senior house officers, and foundation year doctors) and the nursing staff of the orthopaedic ward about their knowledge of pre-operative fasting. A total of nine responses from the doctors and seven responses from the nursing staff were received for the survey. Responses to the survey have been summarised in Table 6. The overall knowledge of pre-operative fasting was found to be inadequate among both the orthopaedic team doctors and the nursing staff, although the doctors demonstrated relatively better knowledge.

**Table 6:** Results of the online survey for orthopaedic team doctors and ward nursing staff

Survey questions	Orthopaedic team doctors		Ward nursing staff	
	Number (n)	Percentage (%)	Number (n)	Percentage (%)
Total number of staff who responded	9	100	7	100
Reasons for fasting before surgery				
To prevent the aspiration of food	9	100	5	71.4
To empty bowels	0	0	1	14.3
To reduce bleeding	0	0	0	0
Avoid high blood sugar	0	0	1	14.3
Do you explain the reasons for fasting to your patients?				
Yes	7	77.8	4	57.1
No	2	22.2	3	42.9
Do all patients requiring general anaesthesia need to be fasted?				
Yes	7	77.8	4	57.1
No	2	22.2	3	42.9
Do all patients requiring regional anaesthesia need to be fasted?				
Yes	2	22.2	2	28.6
No	7	77.8	5	71.4
How long should a patient be fasted prior to surgery?				
6 hours solids and 2 hours clear liquids	9	100	3	42.9
6 hours for both solids and clear liquids	0	0	0	0
Fasting from midnight for both solids and clear liquids	0	0	4	57.1
Overnight fasting	0	0	0	0

## Discussion

The Royal College of Nursing (RCN), which developed the clinical practice guidelines on perioperative fasting in 2005, makes clear recommendations regarding pre-operative fasting time for healthy adults. The guideline recommends that a healthy adult take water and other clear fluids (tea and black coffee without milk, pulp-free juice) for up to two hours prior to the induction of anaesthesia. The guidelines also recommended that the volume of clear fluids have no impact on the gastric residual volume or pH, and therefore, patients can be encouraged to drink unlimited water and clear fluids up to two hours prior to anaesthesia. A minimum fasting time of six hours was recommended for solids and milk for healthy adults prior to anaesthesia. For 'higher-risk' patients with poorly controlled co-morbidities and/or significant gastrointestinal disorders that increase the risk of aspiration, the fasting time should be determined by the anaesthetic team [10]. This guideline was further endorsed by the Royal College of Anaesthetists (RCoA) [10]. The Association of Anaesthetists of Great Britain and Ireland (AAGBI) Safety Guidelines on Pre-operative Assessment and Patient Preparation and the guidelines on pre-operative fasting published by the European Society of Anaesthesiology and the American Society of Anaesthesiologists put forward the same recommendation of two hours of fasting for water and clear fluids and six hours for solids and milk for healthy adults [8,9,12]. Physiologic studies have shown that, in general, clear liquids tend to pass through the stomach within one to two hours, while solids take up to four hours. However, various other factors may affect gastric emptying and cause significant variations in this. Fluids with high osmolality and calories tend to empty slowly. Milk empties at a rate in between clear fluids and solids. The rate of gastric emptying may be slowed by pharmacological agents such as opioids, certain classes of antidepressants, anticholinergics, beta-receptor agonists, and L-dopa. Therefore, it is important to take into consideration these factors when advising on pre-operative fasting [6,7,10].

We observed that there was a significant discrepancy between the recommendations given by the standard guidelines and the current pre-operative fasting practices among the trauma list of orthopaedic patients. The mean fasting time for solids and semi-solids was noted as 15.8 hours, which was significantly higher than the recommended six hours. The longest fasting time for solids and semi-solids was noted as high as 24 hours. The mean fasting time for clear fluids was noted as 10.5 hours, which was more than five times higher than the recommended two hours. It was particularly concerning to note that all the patients in the study population had fasted for more than the recommended six hours for solids and semi-solids, while 98% (n=49) of the patients had fasted for more than the recommended two hours for clear fluids. In addition, only 52% (n=26) had received pre-operative IV fluids, despite the prolonged fasting times observed. A similar study conducted in 2018 in the United Kingdom, involving five National Health Services (NHS) trusts, showed a median fasting time for clear fluids and solids of 5.8 and 16.1 hours, respectively [3]. We noted that the mean fasting time of 15.8 hours for solids found in our study was comparable to the findings in this study. However, the mean fasting time of 10.5 hours for clear liquids noted in our study was significantly higher. A similar study carried out in 2020 in a tertiary hospital in Sri Lanka also yielded comparable findings, with mean fasting times for clear fluids and solids recorded as 7.38 and 12.85 hours, respectively [2].

It is important to note that 64% of the patients in our study (n=32) were over the age of 70. The elderly population has been shown to be more susceptible to dehydration and its adverse effects, including AKI, due to their other co-morbid conditions, polypharmacy, and age-related physiological and psychological factors. Thus, prolonged periods of fasting for clear liquids should be avoided in this population whenever possible [13-15]. In addition, elderly patients with diabetes are at a higher risk of recurrent hypoglycaemic episodes, which can often go unnoticed due to non-specific symptoms and age-related cognitive impairment, which has been shown to worsen their functioning and contribute to frailty [16]. It was noted that 16% (n=8) of the study population had been diagnosed with diabetes mellitus. Therefore, we reiterate the importance of avoiding undue prolonged fasting and hypoglycaemia in such patients.

Many factors may contribute to prolonged fasting, such as changes in the order of the theatre list, difficulty predicting the duration of a surgery, unanticipated prolonged surgeries, unanticipated cancellation of a surgery, not having separate morning and afternoon theatre lists with different times of onset of fasting, and difficulty waking up to eat or drink late in the night or early in the morning. While many of the above factors are hard to control or predict, there are some factors that are still within the control of the medical team that may have contributed to the above findings. The survey conducted among the orthopaedic team doctors and the nursing staff revealed several gaps in their knowledge of pre-operative fasting. Only 42.9% of the nursing staff correctly identified that the pre-operative fasting time should be two hours for clear fluids and six hours for solids/semi-solids. Only 71.4% of the nursing staff recognised the prevention of pulmonary aspiration as the reason for pre-operative fasting. It was seen that 22.2% of the doctors and 42.9% of the nursing staff believed that not all general anaesthetic cases required pre-operative fasting. It is worth noting that 62% of the patients (n=31) had documented instructions to be ‘nil by mouth from midnight’, with no distinction made between clear fluids and solids. There was no distinction made between fasting times for solids/semi-solids and clear fluids in 90% of the patients (n=45). Only 10% of the patients (n=5) had correctly documented fasting instructions as per the standard guidelines. We believe that the lack of adequate knowledge on pre-operative fasting among the personnel responsible for giving the instructions and those responsible for enforcing them may have contributed to this practice.

The American Society of Anaesthesiologists also recommends that all patients awaiting surgery under regional anaesthesia (spinal, epidural, or peripheral nerve block) or procedural sedation should be fasted as for general anaesthesia, as they may require conversion to one during the surgery [9]. Although pre-operative fasting for regional anaesthesia and procedural sedation is not covered by the RCN and AAGBI guidelines, it has been a common recommendation and practice by anaesthetists to fast such patients as for general anaesthesia. This has been the practice in our hospital as well. It was worth noting that 77.8% of the orthopaedic team doctors and 71.4% of the ward nursing staff believed that not all patients undergoing regional anaesthesia required pre-operative fasting.

There were a few cases that needed to be excluded from the study due to unclear documentation of the pre-operative fasting times on the notes or the inability to find the theatre care pathway documents on the online system. The time of last clear fluid and solid that was documented in the theatre care pathway document was based on the patient’s recollection, which at times may not be accurate. We were unable to assess the effects of the prolonged fasting in our audit, as this study was conducted retrospectively. These were some of the limitations that were identified in our study.

## Conclusions

The mean fasting times for clear fluids and solids/semi-solids were 10.5 and 15.8 hours, respectively. These findings were significantly higher than the recommended fasting times of two and six hours for clear fluids and solids/semi-solids, respectively. In addition, only 10% of the patients (n=5) had correctly documented fasting instructions on their notes, as per the standard guidelines. We identified that there were certain gaps in the knowledge of pre-operative fasting among the orthopaedic team doctors and the ward nursing staff.

We recommend the development of a local pre-operative fasting guideline within the hospital, based on the standard guidelines, through collaboration with the anaesthetists, surgeons, and nurses. We suggest that in the departmental induction, all junior surgical doctors should be made aware of the pre-operative fasting guidelines, the reason for fasting, and the effects of prolonged fasting. As it is the nurses that enforce pre-operative fasting in the ward setting, printing out a summary of the pre-operative fasting guidelines, displaying them in the wards, and taking steps to educate the nursing staff about this topic can help address this issue. We also recommend separating the trauma list patients into morning and afternoon lists so that patients who are on the afternoon list can be allowed to have an early breakfast. We have now implemented a 'sip till send' policy in our hospital, where patients are encouraged to sip clear fluids while waiting to be sent for their surgery. We believe that simple steps like these can help to significantly bridge the gap between the recommended fasting times and the actual fasting times of our trauma-list patients.
